# Mechanisms of NO-Mediated Protein S-Nitrosylation in the Lens-Induced Myopia

**DOI:** 10.1155/2022/8296043

**Published:** 2022-11-16

**Authors:** Ying Lu, Weitao Song, Yuanjun Li, Jingge Xiao, Kaixuan Du, Qiuman Fu, Yanni Zhang, Liting Zhao, Yewei Yin, Tu Hu, Dan Wen

**Affiliations:** ^1^Eye Center of Xiangya Hospital, Central South University, Changsha, Hunan 410008, China; ^2^Hunan Key Laboratory of Ophthalmology, Changsha, Hunan 410008, China; ^3^National Clinical Research Center for Geriatric Diseases, Xiangya Hospital of Central South University, Changsha, Hunan 410008, China; ^4^Xiangya School of Medicine, Central South University, Changsha, Hunan 410013, China

## Abstract

**Background:**

Myopia is a chronic ocular disease, emerging as the most common type of refractive error. This study intends to preliminarily explore the roles of protein S-nitrosylation of nitric oxide (NO) in the regulation of myopia by detecting the expression of neuronal nitric oxide synthase (nNOS) and downstream S-nitrosylation, using the animal model of lens-induced myopia (LIM) in mice.

**Methods:**

The 3-week-old C57BL/6 J mice were divided into three groups: group I, lens-induced 0-week group (take eyeballs at the age of 3 weeks); group II, self-control eyes of experimental group (take eyeballs at the age of 7 weeks); and group III, lens-induced 4-week group (take eyeballs at the age of 7 weeks). The diopter and axial length of each group were measured by streak retinoscopes and optical coherence tomography (OCT) before and after model establishment. The protein expressions and locations of nNOS and S-nitrosylated proteins (PSNOs) were measured by western blot and immunofluorescence staining. Site-specific proteomic for protein S-nitrolysation was used to detect the existence and location of S-nitrosylation proteins in the retina of myopic and nonmyopic mice. The Gene Ontology (GO), Kyoto Encyclopedia of Genes and Genomes (KEGG), and motif enrichment analyses were performed. The differential sites were analyzed by GO, KEGG, and motif. Irreversible biotinylation procedure combined with protein purification and western blot was used to detect the protein expression of *α*-enolase (ENO1), a key player in the hypoxia-related signal pathway.

**Results:**

The expressions of nNOS and PSNOs were significantly lower in the retina of experimental eyes than that in self-control eyes and 3-week-old baseline group. A total of 595 S-nitrosylated proteins, 709 S-nitrosylated peptides, and 708 S-nitrosylated sites were identified by site-specific S-nitrolysation proteomics in the retina of myopic and control eyes. A total of 19 differentiation loci were screened, of which 13 sites were downregulated and 6 sites were upregulated in experimental eyes compared with the self-control group. Specifically, the expression of SNO-ENO1 was significantly lower in the retina of experimental eyes than that in self-control eyes and 3-week-old baseline group.

**Conclusion:**

LIM induces the decrease of nNOS and PSNO protein levels in the retina of myopic mice. NO-mediated nonclassical protein S-nitrosylation modification may play an important role in the regulation of lens-induced myopia. ENO1 may be a key factor in the regulation of S-nitrosylation modification of myopia.

## 1. Introduction

Myopia is a chronic ocular disease, as the most common type of refractive error, and the prevalence of myopia is increasing rapidly in recent years [1]. High myopia can cause a variety of serious complications, including higher risk of posterior vitreous detachment, choroidal atrophy, retinal degeneration, retinal detachment, macular hole, and macular hemorrhage [2, 3]. However, the occurrence of myopia is affected by many factors, such as genetic susceptibility and environmental factors. The pathogenesis of myopia is complex, and the mechanism of myopia onset remains unclear. The exploration of the pathological process and pathogenesis is an urgent problem to be solved in the study of myopia.

Nitric oxide, produced by NO synthase (NOS), widely participates in the regulation of nervous system, cardiovascular system, and immune system. NO signaling could be classified into classical and nonclassical schemes. In the classical scheme, normal physiological concentrations of NO acts through activation of its specific receptor soluble guanylyl cyclase (sGC), elevating intracellular 3′,5′-cyclic guanosine monophosphate (cGMP) [4], which serves as a second messenger and activates cGMP-dependent protein kinase (PKG) [5]. Recently, there have been several reports of the participation of non-cGMP-dependent way in cell function regulation. S-nitrosylation, the NO mediated redox-type reversible modification of cysteine thiols, affects the modulation of conformation, stability, and activity of protein and protein-protein interactions [6]. Previous studies have indicated that NO exerted its function via the cGMP dependent pathway and dysregulated NO overproduction caused by inducible nitric oxide synthase (iNOS), enduothelial nitric oxide synthase (eNOS), and nNOS and is critically involved in myopia development [7–9]. However, the mechanism underlying the effect of protein S-nitrosylation in myopia has not been understood.

S-Nitrosocysteine residues often affect protein activity, protein-protein interaction, and protein localization [10, 11]. Numerous studies have shown that dysregulation of NO and S-nitrosothiol (SNO) signaling is involved in progression of many neurodevelopmental, neurobehavioral, and neurodegenerative disorders [12–15]. Under physiologic conditions, protein S-nitrosylation provides protection against further cellular oxidative and nitrosative stress [16]. Nevertheless, the role of protein S-nitrosylation in the development of myopia has not been directly investigated. S-Nitrosoproteomics greatly facilitated the elucidation of multiple underlying processes of protein S-nitrosylation in various species, including mitochondrial fatty acid metabolism, neural signaling, and neurodegeneration [11, 17–19].

In this study, site-specific proteomic profiling of endogenously S-nitrosylated proteins in myopia tissues was performed to provide a new perspective for nitric oxide and protein S-nitrosylation during myopia pathogenesis. The goals of this study were to provide mechanistic insight into the contribution of NO in various ophthalmic diseases, especially in myopia, and suggest potential and promising therapeutic protein targets and sites for treatment.

## 2. Materials and Methods

### 2.1. Experimental Animal

Sixty-five SPF-grade healthy 3-week-old male C57BL/6 J mice weighed 10-15 g were used in this study obtained from the laboratory Animals Department of Central South University. Tropicamide phenylephrine (0.5%) eye drops were utilized to examine the condition of mice eyes, in order to exclude mice with ophthalmic diseases such as keratitis, corneal leukoplakia, pupil atresia, cataract, and vitreous hemorrhage. Mice were given ad libitum access to food and water and reared in a 12 : 12 hr light:dark cycle at approximately 350-500 lux with good ventilation at the temperature of 20°C-25°C, and the cage box and cushion were changed every 3 days to keep the feeding environment clean. All procedures were approved by the Animal Ethics Committee of Central South University (ethical clearance and approval No. 202103175) and adhered to the ARVO Statement for the Use of Animals in Ophthalmic and Vision Research.

C57BL/6 J mice (3-week-old male, *n* = 65) were randomly divided in 3 groups, viz., group I: 15 mice (30 eyes), lens-induced 0-week group (take eyeballs at the age of 3 weeks); group II: 50 mice (50 left eyes), self-control eyes of the experimental group (take eyeballs at the age of 7 weeks); and group III: 50 mice (50 right eyes), -10D lens-induced 4-week group (take eyeballs at the age of 7 weeks).

### 2.2. Establishment of a Model of Optical Defocus Myopia

Use the cyanoacrylate adhesive to adhere the rubber ring and the clear -10D lens periphery (about 8 mm diameter) provided by Kang Ming Company to make an optical defocus lens with a diameter of about 10 mm. Mice were anesthetized with 1% pentobarbital sodium (0.01 ml/g), and the lens was sutured on the skin around the right eye with 4-0 braided polyester sutures thread through interrupted sutures. Hand-made plastic collar with an outer diameter of about 50 mm and an inner diameter of 10 mm was glued to the neck to prevent mice from grabbing off the lens. The periocular skin was disinfected with povidone iodine (0.5 g/L, Annjet) before the operation, and the conjunctival sac was washed with levofloxacin eye drops (3 mg/ml, Baush) to prevent infection. Defocus lenses were checked daily for fit and compliance to keep the optical defocus state. And ocular health was also monitored daily; animals with keratitis, corneal leukoplakia, pupil atresia, cataract, and vitreous hemorrhage were excluded from further analysis. After 4 weeks of induction, the lens was removed, the diopter and the axial length were measured, and the retinal tissue was detected. The left eye served as a paired control and received no treatment.

### 2.3. Diopter and Axial Length Measurement

Refraction and axial length were measured twice for each eye: before and after induction. 1% pentobarbital sodium and 0.5% tropicamide phenylephrine eye drops were applied to the mice eyes to ensure anesthesia and mydriasis, respectively. 10 minutes later, the diopter of the mice was measured using retinal band optometry, and the eye axial length was measured using the Visante OCT (Carl Zeiss Meditec, Germany) by the same experienced optometrist. Each eye was measured three times and averaged.

### 2.4. Determination of Protein S-Nitrosylation

After the measurement of the diopter and axial length, mice were sacrificed via cervical vertebra dislocation, and eyes were subsequently removed. The muscle and fascia tissue was firstly separated, followed by the eyeball being incised along the limbus under the microscope, then the lens and vitreous tissue in the eye were removed, the sclera was separated, and the retina was stored finally at-80°C. The measurement of S-nitrosylated protein of the retina was performed by immunoprecipitation or the biotin switch assay with anti-biotin antibody (Cayman 1 : 1000). The biotin switch assay was performed as described previously by Jaffrey and Snyder [20]. Briefly, the retina tissues were homogenized in the HEN buffer (250 mM HEPES-NaOH, pH 7.7, 1 mM EDTA, 0.1 mM neocuprione) without sodium dodecyl sulfate (SDS), and the MMTS was added to block free thiol groups. Secondly, S-nitrosothiols were reduced by ascorbate, and then the new free thiol groups were biotinylated by biotin-HPDP. The protein with biotin tag was purified by streptavidin. Finally, the biotinylated protein was analyzed by immunoblotting with biotin antibody.

### 2.5. Western Blot

Western blot was performed as described previously [21]. Electrophoresis transferred and blocked was performed in sequence with the protein extracted from the retinal homogenate according to the standard protocol. The primary antibodies, nNOS (Abcam, ab229785, 1 : 1000) and ENO1 (Zen-Bio, R23329, 1 : 1000), were incubated overnight, and the secondary antibody was incubated for 2 hours. The densities of light bands were analyzed quantitatively by ImageJ software.

### 2.6. Immunofluorescence

Another 6 mice in each group were randomly selected, and the eyeballs were fixed in the eyeball fixation at 4°C for 24 hours, followed by the dehydration with graded alcohol. With absorbing water, the eyeballs were transferred to OCT embedding. After frozen in the cryogenic table, retinal tissue was sectioned along the optical axis to a thickness of 15 *μ*m. The section with optic nerve passing through the posterior pole was selected, pasted on the adhesive slides, and stored in the refrigerator at-20°C. Cryosections of each eyeball were frozen in 4% paraformaldehyde for 30 minutes and blocked with 5% normal bovine serum for 1 hour at room temperature. Sections were incubated overnight with primary antibodies specific for nNOS (Affinity, 1 : 100) and washed thoroughly with phosphate-buffered saline. After further incubation with species-appropriate secondary antibodies conjugated to anti-rat 594 (Jackson ImmunoResearch, 1 : 200), sections were counterstained with DAPI (Sigma, Fluoroshield with DAPI), mounted, and photographed using a fluorescence microscope (DM4B, Lecia, Germany).

### 2.7. Site-Specific Identification of S-Nitrosylated Proteins in Retinal Tissue of Myopic Mice

Retinal tissues from experimental eyes and control eyes were collected, and 10 of them were taken as one sample and two biological replicates. A total of 20 mice, 40 eyes, were separately analyzed by site-specific proteomics for discovery of S-nitrosylated proteins. Negative control without sodium ascorbate treatment during biotinylation of S-nitrosylated proteins was included in each experimental and control tissue, which were also analyzed by mass spectrometry to exclude false positive identification due to incomplete blocking. Reliable identifications of S-nitrosylated peptides were finally obtained by searching with MaxQuant (version 2.0.1.0) using a FDR of <1% at both the peptide and protein group levels for control of false identification.

### 2.8. Statistical Analysis

Data were analyzed using SPSS software (version no. 24.0; IBM Corp.). Multiple comparisons were analyzed using ANOVA, followed by Tukey's post hoc test. An independent samples *t*-test was used to analyze independent samples. A paired *t*-test was used to analyze differences between paired experimental specimens. Data were presented as the mean ± SEM. *P* < 0.05 was considered to indicate a statistically significant difference.

## 3. Results

### 3.1. Confirmation of Lens-Induced Myopia

**Figure 1:**
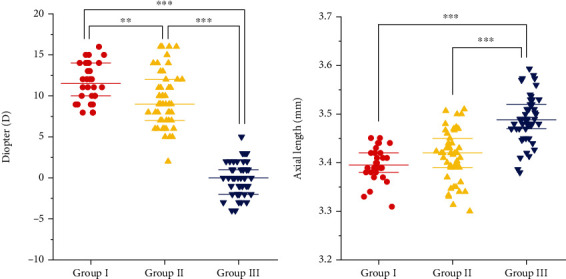
Comparison of refraction and axial length after 4 weeks of LIM. (a) Comparison of refraction. (b) Comparison of axial length (^∗∗∗^*P* < 0.001, ^∗∗^*P* < 0.01, ^∗^*P* < 0.05).

There were no significant differences in refraction or axial length among all groups before the experiment at baseline (*P* > 0.05). Additionally, there were no significant differences in refraction or axial length between the two eyes of the same animal (*P* > 0.05). After 28 days of defocus, refraction for group I, group II, and group III was +11.633 ± 2.385 D, 9.520 ± 3.351 D, and −0.080 ± 1.998 D, respectively (Table 1, Figure 1(a)). After 28 days of defocus, axial length of group I, group II, and group III was 3.397 ± 0.034 mm, 3.415 ± 0.052 mm, and 3.490 ± 0.048 mm, respectively (Table 1, Figure 1(b)).

### 3.2. The Expression of nNOS and PSNO Protein in Retina Decreased in LIM Group

**Figure 2:**
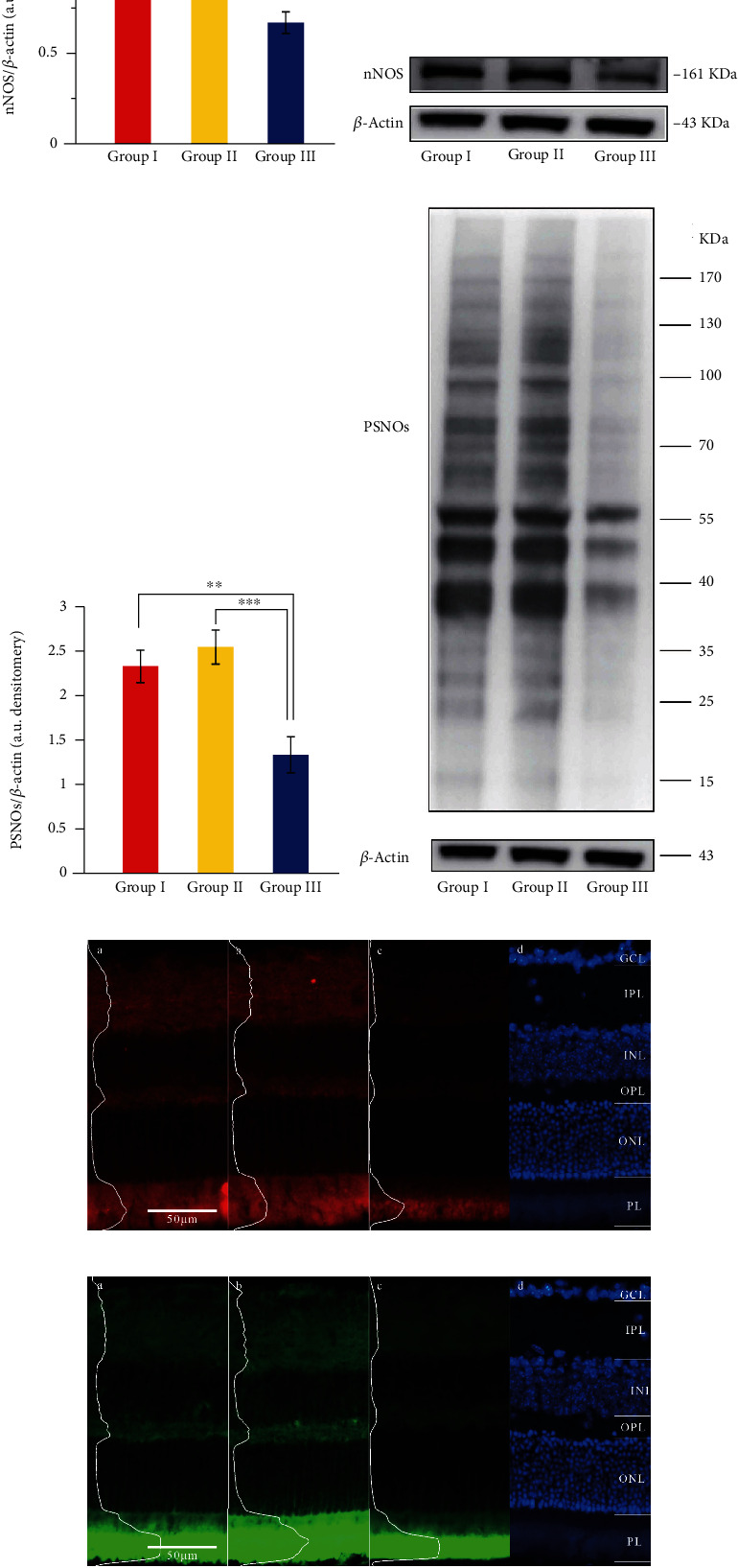
Expression of nNOS and PSNOs in the retina of groups I, II, and III. (a, b) Expression of nNOS in the retina of groups I, II, and III. (c, d) Expression of PSNOs in the retina of groups I, II, and III. (e) The expression of nNOS in the retina of group I, group II, and group III (nNOS: red fluorescence; DAPI: blue fluorescence). (f) The expression of PSNOs in the retina of group I, group II, and group III (PSNOs, green fluorescence; DAPI, blue fluorescence): (a) retina of group I, (b) retina of group II, (c) retina of group III, and (d) DAPI staining nucleus. GCL: ganglion cell layer; IPL: inner plexiform layer; INL: inner nuclear layer; OPL: outer plexiform layer; ONL: outer nuclear layer; PL: photoreceptor layer (^∗∗∗^*P* < 0.001, ^∗∗^*P* < 0.01, ^∗^*P* < 0.05).

The expressions of nNOS in retinal tissues of groups I, II, and III were tested. Abundances of nNOS in group III showed an obvious decrease compared with groups I and II (Figures 2(a) and 2(b)). We further found that the total expression level of PSNOs in group III was also significantly lower than groups I and II (Figures 2(c) and 2(d)). Immunofluorescence showed that the expression of nNOS and PSNOs was high in ganglion cell layer (GCL), inner plexiform layer (IPL), and outer plexiform layer (OPL) but low in inner nuclear layer (INL) and outer nuclear layer (ONL). The expression levels of nNOS and PSNOs in retina in group III were significantly lower than those in groups I and group II (Figures 2(e) and 2(f)). Remarkable downregulation of nNOS expression and protein S-nitrosylation suggested that this NO-mediated protein modification might play a role in myopic pathogenesis.

### 3.3. Site-Specific Identification of S-Nitrosylated Proteins in Retinal Tissue of Myopic Mice

**Figure 3:**
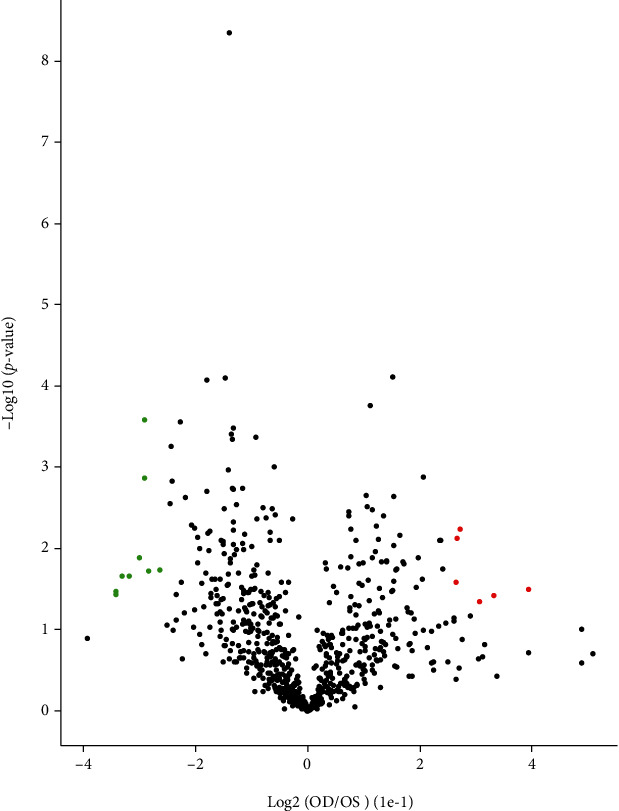
The volcanic map was expressed at the differential site. The green expression is downregulated, the red expression is upregulated, and the black is the differential modification site. The protein S-nitrosytation modification site is derived through LIM versus self-control group.

For a comprehensive view of protein S-nitrosylation, we used a site-specific proteomic approach to characterize S-nitrosylated proteins and modified Cys residues in myopic (group III) and control (group II) retina tissues. In this method, endogenously S-nitrosylated proteins in myopic and control retina were first irreversibly biotinylated via biotin-switch, followed by tryptic digestion, biotin-affinity purification, and final identification of protein identity and modification sites using Orbitrap Exploris TM 480 mass spectrometer. LC-MS/MS analysis was performed on each experimental and control tissues. Biotinylated peptides identified in negative controls were excluded from the corresponding S-nitrosylation dataset. In retinas from four groups, a total of 709 S-nitrosylated peptides and 595 S-nitrosylated proteins were identified. 19 differentially modified modification sites were identified between the myopic and control eyes (fold change < 0.83, *P* < 0.05), of which 13 were downregulated and 6 were upregulated in the myopic eyes compared with the control eyes (fold change > 1.2, *P* < 0.05, Figure 3).

#### 3.3.1. GO Classification and GO Enrichment Analysis

**Figure 4:**
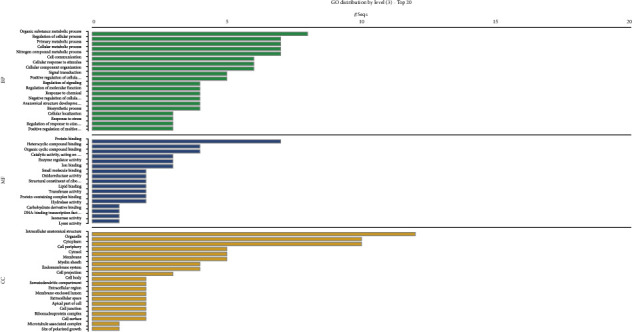
GO classification of differential S-nitrosytation sites in the retina between LIM and self-control group.

**Figure 5:**
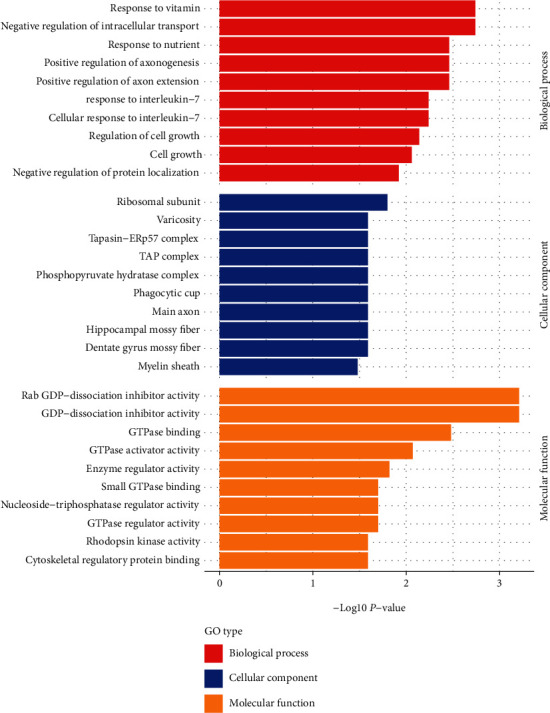
GO enrichment of differential S-nitrosytation site in the retina between LIM and self-control group.

A total of 19 differentially modified modification sites were analyzed by GO classification and GO enrichment analysis (Figures 4 and 5). The results showed that the differential S-nitrosytation modified proteins in the LIM myopia model were mainly related to the following biological processes (BP): cell metabolism (GO: 0044237), cell growth (GO: 0048896, GO: 0016049), signal transduction (GO: 0007165), positive and negative feedback regulation (GO: 0048523, GO: 0051094), response to stress and stimulation (GO: 0050896, GO: 0051716), etc. In myopic eyes, S-nitrosytation protein occurs in different cellular components (CC): cytoplasm (GO: 0016020), nucleus (GO: 0005634), cell membrane (GO: 0016020), ribosome (GO: 0005840), etc. The proteins with differential S-nitrosytation modification in myopic eyes were proved to be mainly related to the following molecular functions (MF): GDP/GTP enzyme activity (GO: 005096, GO: 0005093, GO: 0051020, GO: 0005096), rhodopsin kinase activity (GO: 0050254), etc.

#### 3.3.2. KEGG Pathway Analysis

**Figure 6:**
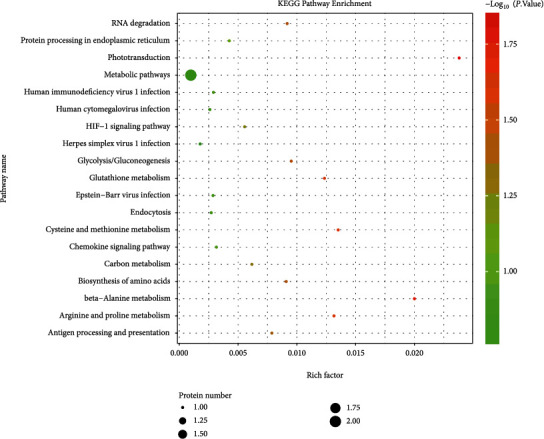
Enrichment analysis of S-nitrosytation KEGG pathway in the retina between LIM and self-control group.

In order to better understand the biological role of protein S-nitrosytation modification in the pathogenesis of myopia, the KEGG pathways of identified 19 differentiated sites were further analyzed (Figure 6). Analysis showed that S-nitrosytation sites were enriched in signal pathways related to protein processing (mmu04141), glycolysis/gluconeogenesis (mmu00010), photoconduction (mmu04744), and HIF-1 (mmu04066).

#### 3.3.3. Motif Enrichment Analysis

**Figure 7:**
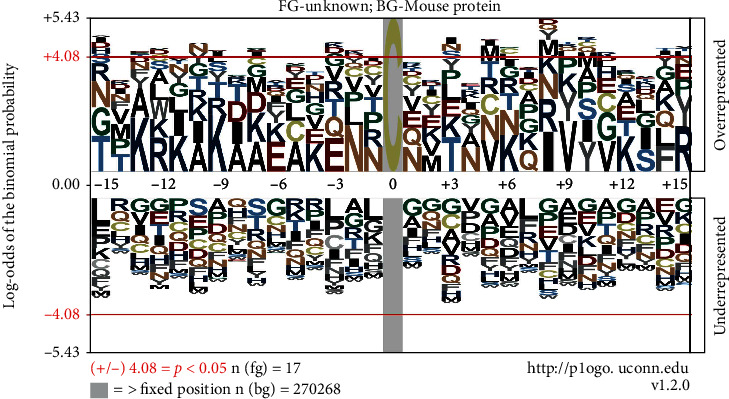
Motif analysis of differential sites of S-nitrosytation in the retina between LIM and self-control group.

Previous studies have shown that the amino acid compositions on both sides of the cysteine residue have a great influence on the sensitivity and specificity of the redox-mediated posttranslational modification of cysteine [3]. In order to further understand the environmental driving factors of protein, the amino acid composition characteristics near the S-nitrosytation residues of 19 differential sites in the retina of groups II and III were analyzed by using the Motif-X algorithm (Figure 7). The result revealed that the amino acid residues containing basic and acidic side chains near the S-nitrosytation cysteine residues were highly expressed, such as basic amino acid lysine (K) and acidic amino acid glutamic acid (E). It is suggested that the acid-base sequence may play a potential role in promoting the pathological process of myopia mediated by redox modification.

### 3.4. The Expression of ENO1 and SNO-ENO1 in Retina of LIM Group Showed Differential Changes

**Figure 8:**
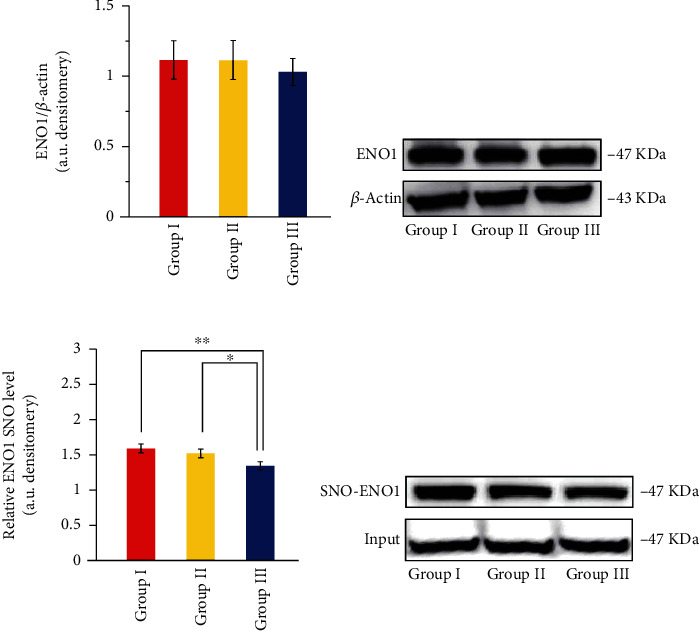
The expression of ENO1 and SNO-ENO1 was detected in the retina of groups I, II, and III. Input was used as the positive control group, and the existence of ENO1 protein in the cell lysate was verified.

Hypoxia has been shown to be an important mechanism of myopic pathological injury in recent years. ENO1 is the key enzyme in the last step of glycolysis pathway, which is closely related to tissue ischemia and hypoxia. The analysis of differential sites found that the expression of SNO-ENO1 was downregulated in the retina of myopia. In order to explore the role of ENO1 in the pathogenesis of myopia, the expressions of ENO1 in the retina of groups I, II, and III were detected by immunoblotting. After 4 weeks of modeling, there was no significant difference in the expression of ENO1 protein among the three groups (*P* > 0.05, Figure 8(a)). Lens induced did not change the protein expression level of ENO1.

In order to explore whether ENO1 participates in the regulation of myopia by S-nitrosytation, the expression levels of SNO-ENO1 in the retina of groups I, II, and III were further detected, and the expression level of ENO1 in the same sample was used as a reference. It was found that the expression level of SNO-ENO1 in group III was significantly lower than that in group I (*P* = 0.006, Figure 8(b)) and the left eye in group II (*P* = 0.036, Figure 8(b)). There was no significant difference in the expression of nNOS between group I and group II (*P* = 0.378, Figure 8(b)). Lens induced decreased the expression of SNO-ENO1.

## 4. Discussion

Myopia, especially high myopia, as a chronic ophthalmopathy with high incidence, has caused a huge social and economic burden. Complications such as posterior vitreous detachment, choroidal atrophy, retinal degeneration, retinal detachment, macular hole, and macular hemorrhage caused by high myopia are the main causes of blindness [2, 3]. At present, it is believed that the changes of signal pathway caused by abnormal visual stimulation play an important role in the occurrence and development of myopia. There are many studies focusing on signal pathways related to the myopia. NO is known to participate in the regulation of myopia through the classical cGMP signal pathway [7, 22–24]. Fujii et al. found that form deprivation reduced the iNOS mRNA expression in chick retina-RPE-choroid [22]. Another study showed that after form deprivation 7 days, the activity of NOS in the deprivation group was lower than in the control group, but after form deprivation 14 days and 21 days, the activity of NOS in the deprivation group increased rapidly and significantly higher than in the control group. This trend of decreasing first and then increasing may be a way of regulating myopia through inducing different responses of eNOS and nNOS by acute and chronic hypoxia [7]. Additionally, the results of our previous studies on form deprivation in guinea pigs for 1 week, 2 weeks, and 3 weeks showed that with the extension of form deprivation time, the expression levels of nNOS and cGMP gradually increased in comparison with the control group, and there was a positive correlation between them [23, 24].

In this study, we showed that after 4 weeks of lens-induced, the expression level of nNOS in the retina of myopic mice was significantly lower than the control group, which was contrary to the previous research results. We speculate that this discrepancy might be due to the differences in animal model, model selection, and modeling time. Since LIM and from deprivation myopia (FDM) belong to two different myopic models in terms of pathogenic mechanism, the expression of NO is likely to be distinct.

We also conjecture that NO may play different roles in different stages of myopia development and may dynamically regulate the occurrence and development of myopia based on the variance of NOS expression at different time points. In the follow-up research, further investigation like detecting NOS at multiple time points in the LIM model should be done to better clarify the role of NO in the occurrence and development of LIM.

Previous studies of NO mainly focused on the classical cGMP-related signal pathways, especially in the nervous system, and NO is known to activate important physiological cascade responses and participate in the regulation of neuronal differentiation and synaptic plasticity in the central nervous system [4]. Studies have shown that synaptic plasticity of NO and cGMP in various brain regions of hippocampus, cerebellum, cerebellum, and striatum is necessary, and NO-cGMP-related signal pathways have a crucial role in long-term potentiation (LTP), long-term depression (LTD), and learning [25]. Impaired NO and cGMP signaling are closely related to the etiology and progression of neurodegenerative diseases and cognitive impairment [26–28]. In recent years, the role of NO-mediated S-nitrosytation modification in diseases has received more attention. Many diseases such as atherosclerosis, malignant tumor, Alzheimer's disease, Parkinson's syndrome, neuronal degeneration, and Huntington's chorea are characterized by abnormal protein S-nitrosytation products [29]. Some researchers have also studied the regulation of NO-mediated S-nitrosytation on the retina: Vielma et al. found that the amplitude of ERG in rats unrelated to cGMP was increased by vitreous injection of exogenous NO donors, and then NO donors caused the increase of GCL and PL S-nitrosytation protein through S-nitrosytation immunofluorescence experiments. It was suggested that NO may improve retinal function by S-nitrosytation modification [30]. In another study of light and light withdrawal, Bloom found that S-nitrosytation protein signals were strong in the retinas of light-adapted mice, while there was almost no S-nitrosytation signal in dark-adapted retinas, proving that the S-nitrosytation of retinal tissue is light-dependent. Taken together, these studies highlight the wide presence of nitroso-modified proteins in retinal tissue and suggest that myopia is closely related to signals including light intensity and light conduction, which can be influenced by experimental myopia model. However, previous studies have not been able to confirm the role of NO-mediated S-nitrosytation modification in the pathogenesis of myopia. In this study, we found that PSNOs in myopic retina were significantly lower than the control group, and immunofluorescence showed the expression of PSNOs high in GCL, IPL, and OPL layer but low in INL and ONL layer. These findings indicate that the decrease of the PSNO expression in the retina of myopia may be related to the decrease of light stimulation in myopic models.

We identified 19 differential modification sites through site-specific identification of S-nitrosylated proteins. Using bioinformatics analysis, we found that the differentially modified proteins were enriched in the signal pathways related to the occurrence and development of myopia, such as energy metabolism, photoconduction, and HIF-1. Thus, we hypothesize that NO is involved in the occurrence and development of myopia through S-nitrosytation modification.

Differential site analysis showed that compared with the control group, the expression of SNO-ENO1 in HIF-1 signaling pathway was downregulated. Studies have shown that hypoxia-induced metabolic reprogramming from mitochondrial respiration to glycolysis required the participation of ENO1, which is a key enzyme in glycolysis [31]. ENO1 has also been reported to be associated with hypoxic-ischemic retinal disease. The expression of HIF-1 and ENO1 in human retinal pigment epithelial cells significantly upregulated under hypoxia and anoxia. In subsequent experiments, it was found that silencing ENO1 did not affect the content of hypoxia-induced vascular endothelial growth factor (VEGF), suggesting that ENO1 may be involved in the regulation of hypoxia pathways other than VEGF [32]. Hypoxia injury is an important mechanism of myopia pathological injury proposed in recent years. Previous studies related to myopia hypoxia were mainly concentrated in the sclera, considering that sclera hypoxia was the trigger factor of myopia [33, 34]. The results of mass spectrometry and immunoblotting showed that there was no significant difference in the expression of ENO1 between myopic and nonmyopic mice, but SNO-ENO1 was downregulated in myopic retina. Recent studies have shown that hypoxia is the key mechanism of myopia, and ENO1 is closely related to hypoxic ischemic retinal disease. We speculate that ENO1 may mediate the occurrence and development of myopia through the dynamic regulation of S-nitrosytation modification and denitrification modification in the hypoxia signal pathway. We further suggest that SNO-ENO1 may be an inactivated form of ENO1. When the tissue is in S-nitrosytation state, ENO1 exists in the state of S-nitrosytation modification, while when the tissue is anoxic, SNO-ENO1 is denitrified to activate ENO1 and participate in the process of glycolysis. However, the research to date has not been able to elucidate of the mechanism of action of the protein around the world, which is worthy of further research and exploration, especially in various ischemic and hypoxic diseases and the occurrence and development of myopia.

## 5. Conclusion

Considering all of this evidence, it seems that NO participates in the occurrence and development of myopia through S-nitrosytation modification, and ENO1 regulates this process as a target protein of S-nitrosytation modification. A large number of endogenously S-nitrosylated proteins and their modification sites in C57BL/6 J mouse retinal tissue were identified by site-specific proteomics. A total of 19 differentiation loci were screened, of which 13 sites were downregulated and 6 sites were upregulated in experimental eyes compared with the self-control group. These differentiation loci involving multiple processes and signaling pathways such as phototransduction, hypoxia, and energy metabolism are closely associated with myopia. These findings provided novel insights into NO signaling and S-nitrosylation in myopia development and also a basis for the diagnosis and treatment of retinal diseases based on protein modification.

## Figures and Tables

**Table 1:** The refraction and the axial length in 4 weeks after modeling ($\bar{x}\pm s$).

	Group I	Group II	Group III	*P*_I−II_	*P*_I−III_	*P*_II−III_
Diopter/D	11.633 ± 2.385	9.520 ± 3.351	−0.080 ± 1.998	0.003	<0.001	<0.001
Axial length/mm	3.397 ± 0.034	3.415 ± 0.052	3.490 ± 0.048	0.345	<0.001	<0.001

## Data Availability

The datasets used or analyzed during the current study are available from the corresponding author on reasonable request.
